# High‐Yield Deterministic Focused Ion Beam Implantation of Quantum Defects Enabled by In Situ Photoluminescence Feedback

**DOI:** 10.1002/advs.202300190

**Published:** 2023-04-23

**Authors:** Vigneshwaran Chandrasekaran, Michael Titze, Anthony R. Flores, Deanna Campbell, Jacob Henshaw, Andrew C. Jones, Edward S. Bielejec, Han Htoon

**Affiliations:** ^1^ Center for Integrated Nanotechnologies Materials Physics and Applications Division Los Alamos National Laboratory Los Alamos NM 87545 USA; ^2^ Sandia National Laboratories Albuquerque NM 87123 USA; ^3^ Center for Integrated Nanotechnologies Sandia National Laboratories Albuquerque NM 87123 USA

**Keywords:** focused ion beam, in situ photoluminescence, quantum defects, silicon carbide, single photon sources

## Abstract

Focused ion beam implantation is ideally suited for placing defect centers in wide bandgap semiconductors with nanometer spatial resolution. However, the fact that only a few percent of implanted defects can be activated to become efficient single photon emitters prevents this powerful capability to reach its full potential in photonic/electronic integration of quantum defects. Here an industry adaptive scalable technique is demonstrated to deterministically create single defects in commercial grade silicon carbide by performing repeated low ion number implantation and in situ photoluminescence evaluation after each round of implantation. An array of 9 single defects in 13 targeted locations is successfully created—a ≈70% yield which is more than an order of magnitude higher than achieved in a typical single pass ion implantation. The remaining emitters exhibit non‐classical photon emission statistics corresponding to the existence of at most two emitters. This approach can be further integrated with other advanced techniques such as in situ annealing and cryogenic operations to extend to other material platforms for various quantum information technologies.

## Graphical abstract

Defects in solid state materials are actively pursued for various quantum information technologies. However, high yield deterministic implantation remains a major challenge. Here nanometer resolution focused ion beam and in situ PL inspection are used iteratively to demonstrate near unity yield implantation of an array of *silicon vacancy defects* in 4H‐silicon carbide.

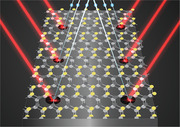

## 1 Introduction

The last decade has witnessed a dramatic expansion in the field of defect driven quantum information science, where solid state quantum defects mimicking the coherence properties of trapped ions are exploited for storing, processing and transducing of information as well as sensing physical phenomena beyond classical detection limits.^[^ ^1^ ^]^ The paradigm of this research field has also shifted from building proof of principle devices around a serendipitous discovery—natural defects in solids—toward deterministically placing defects of desired properties into prefabricated photonic/electronic integrated circuits.^[^ ^2^ ^]^ To this end, focused ion beam (FIB) implantation becomes the tool of choice for placing ions and defects with tens of nanometer (nm) spatial precision.^[^ ^1^, ^3^ ^]^ However, the yield for successful creation of functional defects demonstrated to date via FIB implantation remains relatively low for industry scale fabrication of quantum devices. This low yield stems mainly from two factors: 1) though FIB allows placement of ions with nm scale resolution, the number of ions implanted is still governed by Poissonian statistics; 2) only a small fraction of the ions implanted exhibit desired optical characteristics (e.g., single photon purity). While recent development to in situ ion counting approach provides an effective solution on the former,^[^ ^4^ ^]^ the latter remains standing as a major challenge.

Among the wide variety of quantum defects and their hosts, silicon vacancy defects *V*_Si_ in silicon carbide ( SiC) have emerged as one of the most promising material system: because *V*_Si_ not only exhibit electronic spin fine structure and long spin–coherence times highly desired for quantum sensing and spin–qubit applications, SiC host is also perfectly compatible with CMOS fabrication technology for integration of *V*_Si_ into quantum photonic platforms.^[^ ^5^ ^]^ Key quantum functionalities such as high‐fidelity spin initialization,^[^ ^6^ ^]^ indistinguishable photon emission,^[^ ^7^ ^]^ and integration into p‐i‐n junction for electrical read out of spin state^[^ ^8^ ^]^ have been recently demonstrated with *V*_Si_. The key to push forward this important material system to a scalable platform relies on the deterministic creation of *V*_Si_ defect centers and therefore FIB method stands out from the numerous attempted approaches including electron irradiation,^[^ ^7^, ^9^ ^]^ neutron irradiation,^[^ ^10^ ^]^ laser writing,^[^ ^11^ ^]^ proton beam writing,^[^ ^12^ ^]^ and ion implantation.^[^ ^13^ ^]^ Due to the Poissonian distribution in numbers of implanted defects and low defect activation yield using FIB, the best of these efforts to date can only yield a maximum of ≈35% of the targeted spots with quantum emitters while more than 8% of the targeted spots remain unfilled (**Table** **1**), revealing a requirement for significant improvement. Here, we present an in situ single defect photoluminescence (PL) feedback assisted FIB implantation of *V*_Si_ in 4H‐SiC. By performing low dose implantation and widefield single defect PL imaging in a loop with removal of the implantation target after detection of single defect luminescence, we are able to demonstrate creation of a single quantum emitter with 70% yield and filling 100% of the targets with no more than 2 emitters. Our approach can readily be scaled up and automated to create a large array of quantum devices. In situ laser annealing capability can also be added for defects requiring high‐temperature activation.

**Table 1:** Single *V*_Si_ defect creation in 4H‐SiC

Ref	Method	Source	Energy	Depth	1‐Defect in an array (as implanted)	1‐Defect in an array (post‐treat)	Unfilled spots	Emitter number range
[7]	Broad electron	e^−^	2 MeV	–	–	–	–	–
[10]	Broad neutron	*n* ^0^	0.18–2.5 MeV	–	–	–	–	–
[11]	Laser writing	–	NA	Deep (40 µm)	≈30%	No need	30%	0–5
[12a]	Proton beam writing	H^+^	1 or 2 MeV	Deep (10 or 30 µm)	<10%	No need	–	max 20
[13a]	Mask and broad ion	He^+^	6 keV	Shallow (54 nm)	–	–	–	–
[13b]	Mask and broad ion	C^+^	20 keV	Shallow (50 nm)	≈34%	≈32%	10%	0–6
[13c]	Mask and broad ion	C^+^	30 keV	Shallow (40 nm)	≈34%	No need	15%	0–4
[13d]	FIB	He^+^	30 keV	Deep (179 nm)	–	≈35%	20%	0–10
[13e]	FIB	Li^+^	100 keV	Deep (370 nm)	–	≈7%	8%	0–18
This work	FIB (in situ filling)	Li^+^	80 keV	Deep (307 nm)	≈70%	No need	0%	1 or 2

## 2 Results

### 2.1 FIB Implantation with In Situ Photoluminescence Feedback

**Figure 1:**
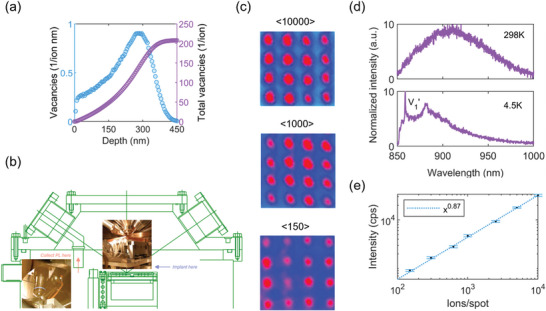
a) Vacancy creation as a function of depth for a 80 keV Li^+^ beam. The small amount of vacancies per depth aids in the creation of single isolated *V*_Si_. b) Schematic of the setup showing the positions for FIB implantation and in situ PL measurement. Since the two locations are spatially separated, a high‐resolution stage is used for sample positioning. c) Normalized color scale widefield PL images collected immediately after implantation using a heavy ion dose of <10 000>, <1000>, and <150> ions per spot. Entire 4 × 4 array is filled with bright spots. Pitch is 2 µm. d) Room and cryogenic temperature spectra of implanted spot show creation of *V*_Si_ defect center. e) Average PL intensity counts of bright spots have a near linear relation with the input implantation doses greater than <100> ions per spot. The error bars denote the standard deviation between all investigated points.

Ion implantation is performed using an A&D nanoImplanter (nI), a 100 kV FIB system, which provides <50 nm targeting resolution after an alignment performed over four fiducial marks within the 200 µm ion beam writefield.^[^ ^14^ ^]^ An example of the achievable targeting resolution is shown in Figure S1 (Supporting Information), while the on‐chip fiducial marks are shown in Figure S2 (Supporting Information). This sub‐50 nm targeting resolution is close to the current state‐of‐the‐art of FIB for liquid metal alloy ion source (LMAIS), mostly limited by chromatic aberrations due to the relatively large energy spread here compared to elemental ion sources such as galliumand helium. We utilize a LMAIS made of Au_66_Si_15_Li_19_ with a Wien filter to pick out lithium ions (7‐Li^+^) for implantation^[^ ^15^ ^]^ as they are shown to minimize damage clusters along the track and total damage. According to Stopping and Range of Ions in Matter (SRIM) simulations, Li^+^ ions have a landing energy of 80 keV at 80 kV acceleration potential with a range of 307 ± 66 nm in SiC. 69.5 keV of ion energy is lost to electronic stopping not leading to creation of a vacancy and remaining 10.5 keV is lost to nuclear stopping, leading to creation of either a vacancy or a replacement collision. The number of vacancies created by the ion beam is shown in **Figure** **1**a. Since only about 0.9 vacancies are created per nm, the likelihood of creating a single *V*_Si_ defect along the Li^+^ track is increased over the use of a heavier ion such as silicon. The ion beam was pulsed such that pulse length is set based on a beam current measurement at a Faraday cup on the sample holder to contain0, a targeted average number of ions.

An in situ PL imaging setup is built into the nI as schematically shown in Figure 1b. Due to the enclosed ion beam columns of the nI (Figure 1b, inset), we perform PL imaging and FIB implantation at two different locations indicated by blue and orange lines. After the FIB implantation at the targeted locations, the stage is moved toward the PL inspection point and brought to the focal plane of our objective microscope for PL inspection (Figure 1b, inset). A widefield PL imaging method is employed to quickly check the successful creation of defects right after the implantation. As our widefield field of view is around 10 µm, we aim to create a 4 × 4 spots with 2 µm pitch target array of defect centers. We first demonstrate the sensitivity of our integrated PL system by showing the widefield PL images collected immediately after just 1 cycle of heavy dose implantation of <10 000>, <1000>, and <150> ions per spot in Figure 1c. We observe the entire 4 × 4 arrays are filled with bright spots. We spectrally resolve the bright spots at room and cryogenic temperatures externally, shown in Figure 1d, that demonstrates successful creation of *V*_Si_ defect centers in 4H‐SiC matching the *V*_1_^'^ emission at 858 nm from previous reports.^[^ ^16^ ^]^ We are able to fill the entire 4 × 4 arrays with such bright spots by using input implantation doses greater than <100> ions per spot in just 1 cycle of implantation. From the observation of a near linear relation between the average PL intensities and the input implantation doses till <10 000> ions per spot as shown in Figure 1e, we can conclude that the mean number of emitters reduces linearly with the number of implanted ions per spot. Here we find the standard deviation of PL intensities only slightly vary, from a 5.3% at <10 000> ions per spot to 4.5% at <150> ions per spot, upon reducing the input implantation doses.

**Figure 2:**
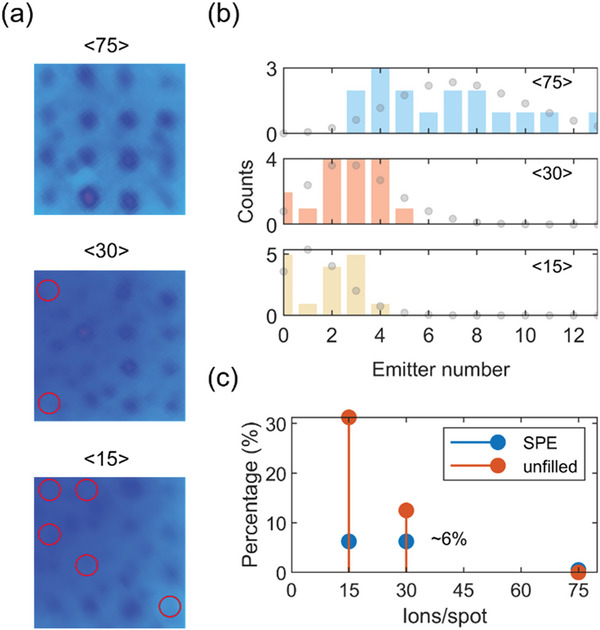
a) Normalized color scale widefield PL images collected immediately after 1 cycle of implantation using lower ion doses of <75>, <30>, and <15> ions per spot. Pitch is 2 µm. Red circles indicate unfilled spots. Variations in the PL emission intensities at different bright spots indicate the number of emitters is different at those spots. b) Histogram of emitter numbers for indicated implantation doses. Gray dots are the expected Poisson distribution of emitters assuming a 10% activation yield. c) SPE yield of ≈6% is observed for lower ion doses of <30> and <15> ions per spot. However, the unfilled spots increase for lower ion doses.

Upon further reducing the input implantation doses lesser than <100> ions per spot as shown in **Figure** **2**, we start noticing 3 key points for just 1 cycle of implantation: 1) variances in the PL emission intensities at different bright spots indicate the number of emitters is different at those spots, 2) 4 × 4 arrays are not completely filled, 3) single photon emitter yield is very low. We explain these observations by showing results for <75>, <30>, and <15> ions per spot in Figure 2. First, we show the normalized color scale widefield PL images for these lower ion doses in Figure 2a. While the entire array is filled with bright spots for <75> ions per spot, we observe a wider variation in PL intensity counts at these spots. The same variations are observed for <30> and <15> ions per spot with the additional observation of unfilled spots. By performing second‐order autocorrelation measurements on these array spots ex situ, we obtain just 1 spot out of 16 locations to be a single photon emitter (SPE) amounting to a ≈6% yield for <30> and <15> ions per spot implantations, while no SPE at <75> ions per spot is observed. We take an average SPE brightness count of 800 counts/second as a base factor and estimate the number of emitters created at the targeted locations which is shown as a histogram in Figure 2b. The expected emitter number distribution based on a Poisson process assuming a 10% activation yield is shown as gray dots in each of the three panels. The standard deviation in the Poisson process is √*N*, where *N* is the number of emitters. While this closely matches the observed emitter distribution for <75> and <30> ions per spot, we notice a deviation for <15> ions per spot due to the simultaneous increase in the unfilled number of spots.

Our results demonstrate that the use of several tens of ions per spot for input implantation would result in a larger emitter number distribution and further lowering of the input implantation dose could help narrow this distribution but at the same time would increase the number of unfilled spots. This shows that to create a scalable number of single defect centers through FIB, it is required to lower the input ion dose. Even then, just a 1‐cycle implantation would result in uncontrolled defect number creation (we created anything between 0‐to‐4 emitters by using 1‐cycle of <15> ions per spot) with the lower probability of single defect center (6% here). Overall, we notice the single photon emission can only be observed for lower ion doses. But this also simultaneously results in an increase in unfilled spots as we reduce the input implantation dose, from a ≈12% for <30> ions per spot to a ≈31% for <15> ions per spot. Therefore, using FIB with just 1 cycle of implantation results in a probabilistic creation of SPE with less than 10% success rate.

### 2.2 In Situ Filling of an Array

**Figure 3:**
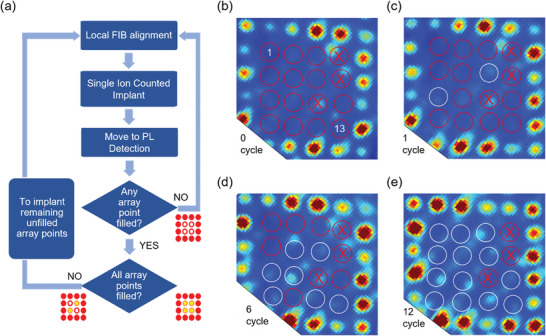
a) Process flow for creating single defects using FIB by getting feedback from our integrated in situ PL system. b) Implant location with pristine center area. Red circles indicate the 13 target locations. Implantation avoided at locations marked with crossed circles that displayed a natural fluorescing background. c) Implant location after one cycle of implantation by <3> ions per spot. Two locations marked with white circles are identified as successfully created defect centers. d) A total of 7 emitters are identified at the end of 6 cycles. e) All 13 target locations are completely filled with emitters at the end of 12 cycles. Pitch is 2 µm.

To advance from this random creation of emitters towards fully deterministic implantation of SPEs at pre‐defined positions, we use our in situ optical characterization as a feedback mechanism to check the creation of defect by investigating the PL on the implanted spot and repeating the implantation for multiple cycles on the same unfilled spots until we observe the bright spot on the pre‐defined positions. **Figure** **3**a shows the process flow for creating single defects using FIB by getting feedback from our integrated in situ PL system.

To perform in situ filling of the array, we pulse the ion beam such that each location obtains an average of 3 ions at each cycle. Prior to performing in situ filling of lower ion doses, an array of high brightness emitters is created through implantation of <500> ions around the region of interest as a local landmark. The widefield PL image as recorded in the in situ PL setup showing the alignment pattern is shown in Figure 3b. The center area of the array is left empty for later in situ PL. We avoid implanting at 3 locations marked by crossed circles in Figure 3b due to natively occurring background emission. Therefore, we choose 13 target locations for in situ array filling marked as red circles in Figure 3b.

After 1 cycle of implantation of <3> ions into each spot, a widefield PL image is taken (Figure 3c). We observe two spots brighten up at locations 6 and 7 while the rest are still unfilled. We then proceed with the next cycle of implantation of <3> ions per spot, but the two white circles denoting newly formed emitters at the end of 1st cycle are excluded from further implantation to target implanting only the remaining unfilled spots. This cycle of implantation and PL inspection are repeated one after another. The targeted implantation on an entire array can be done at once, without any need of optical mask procedures, by simply adding or removing the pre‐defined target spots in lithography software. Each cycle only takes about 10 minutes comprising: 2 minutes for implantation, 5 minutes for stage movement followed by PL collection, and 3 minutes for analysis followed by stage movement. Figure 3d shows the partially filled array with 7 locations brightening up after 6 cycles of implantation. This process is repeated until all the 13 target locations are filled. Figure 3e shows the fully filled array of emitters which in total took 12 cycles of implantation. The whole process only requires <2 hours.

## 3 Discussion

**Figure 4:**
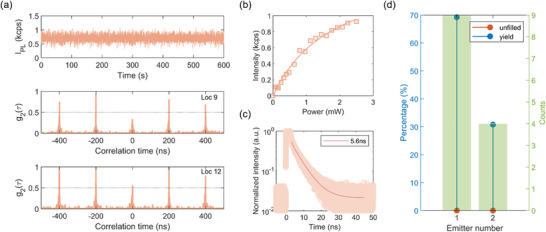
a) (top) A stable emission intensity is obtained for 600 s. (middle) Second‐order autocorrelation *g* ^2^(*τ*) collected at location‐9 shows a single photon emission with *g* ^2^(0) = 0.24. (bottom) Location‐12 shows *g* ^2^(0) = 0.52 indicating an emission from at most 2 quantum emitters. b) PL intensity of single *V*_Si_ defect center saturates upon increasing the input excitation power. c) PL lifetime of 5.6 ns is obtained. d) Unfilled spots at 13 locations are zero using in situ PL feedback. SPE yield of ≈70% is obtained from those filled spots with the remaining spots being at most 2 defect centers.

After filling all implant locations via in situ PL, the sample is taken out of the vacuum chamber to enable further measurements. **Figure** **4**a shows a representative example for the background corrected PL intensity time‐trace recorded from the created bright spot at location‐9 that displays a stable emission over 600 s. Upon checking the second‐order autocorrelation function at room temperature on the filled 13 spots after background correction, we find that 9 of them show single photon emission with *g* ^2^(0) < 0.5. We show an example of SPE with *g* ^2^(0) = 0.24 from location‐9 in the middle panel of Figure 4a. The number of emitters created can be predicted by estimating *g* ^2^ (0) = 1 − 1/*n* for *n* number of emitters.^[^ ^17^ ^]^ It is expected that *g* ^2^(0) = 0 for 1 emitter, *g* ^2^(0) = 0.5 for 2 emitters, *g* ^2^(0) = 0.67 for 3 emitters, and so on. We attribute the non‐zero *g* ^2^(0) values in those 9 spots to the background noise. This background influence of *g* ^2^(*τ*) traces is widely reported in most of the localized defects in semiconductors such as SiC,^[^ ^13c,e^ ^]^ color centers in diamond,^[^ ^17b^ ^]^ hBN,^[^ ^18^ ^]^ GaN,^[^ ^19^ ^]^ and transition metal dichalcogenide^[^ ^20^ ^]^ to name few. A representative example for a remaining location containing at most 2 quantum emitters is shown in the bottom panel of Figure 4a that displays a *g* ^2^(0) in the range of 0.5–0.7. To compare the similar optical properties reported for single *V*_Si_ defect centers in 4H‐SiC,^[^ ^16^ ^]^ we show PL intensity saturation in Figure 4b and PL lifetime of about 5.6 ns in Figure 4c.

These measurements and analysis show that we can fill 9 out of the 13 targets with single quantum emitters. *g* ^2^(0) of the remaining 4 locations indicate no more than 2 emitters. See Figure S3 (Supporting Information) for *g* ^2^(*τ*) plots of all 13 targets. While prior reports indicate that annealing at 400 °C for 2 h could clean up the background emission,^[^ ^13e^ ^]^ we did not observe any improvement in our current sample. While we intend to create only 1 emitter per spot, the explanations for *g* ^2^(0) > 0.5 at 4 targets are that the large background present in our sample overlapping with the emission from the quantum emitter could have led us to wrongfully classifying a single emitter as a double emitter. Another possible explanation of *g* ^2^(0) > 0.5 is that we performed assessment of the implantation by eye, hence due to human error we may have not stopped implantation upon creation of a single emitter but reimplanted the same site until two emitters were formed in the subsequent cycles and only stopped implantation then. Lastly, since the laser used in autocorrelation measurements had an excitation spot size of 1 µm, and emitters are spaced by only 2 µm, there is the potential of collection of PL from a weakly excited emitter at an adjacent array location which might also lead to *g* ^2^(0) > 0.5. The ≈70% SPE yield with no unfilled spots (Figure 4d) demonstrated here is among the state‐of‐the‐art value. We believe the FIB scaling up of larger quantum emitter arrays in solid‐state materials would be made possible in a much more efficient manner by our iterative in situ PL approach.^[^ ^21^ ^]^ Our experiment could be further improved by having a SiC substrate with lower background emission, engineering of photonics devices for enhanced PL collection, and possibly integrating a machine learning algorithm to automatically detect the creation of defect by checking the target for any change in PL counts.

## 4 Conclusion

This experiment overall clearly shows that our in situ PL assisted FIB implantation overcomes two fundamental technical barriers that hinder FIB from achieving fully deterministic implantation, namely Poissonian distribution in number of implanted ions and only a small fraction of implanted ions exhibiting desired single photon emission properties. Our achievement of implanting 100% of the targeted location with 2 or less and 70% of the location with SPEs confirmed by second‐order autocorrelation experiment represent an order of magnitude improvement over single pass FIB implantation at optimum ion dose (6% from this experiment shown in Figure 2 and 7% from ref. [13e]). These numbers also represent at least a factor of 2 improvement over the best implantations reported to date (≈35% shown in Table 1 with a caveat of random creation of 0 to 10 emitter numbers). Complete elimination of unfilled spots demonstrated here could also bring a significant cost reduction for implantation into prefabricated photonic/electronic devices. Currently, our experiment can be performed in a reasonable time of <2 hours and readily be scaled up to perform implantation of large arrays in multiples of 1‐emitter through automation. By simply coupling a high‐power laser to the in situ PL system, we could add in situ annealing capability to the sample allowing improvement of emitter optical properties and open the possibility of extending this powerful deterministic defect implantation strategy to other material platforms such as diamond^[^ ^22^ ^]^ and hBN.^[^ ^23^ ^]^ Both diamond and hBN have successfully demonstrated creation of FIB created quantum emitters, however, they currently require a high‐temperature annealing step which is not integrable into the FIB system. Through the use of laser annealing, the needs of the wider quantum information science research community can be addressed. Specifically, the in situ PL with in situ laser annealing may revolutionize the fabrication of diamond atomic force microscope (AFM) tips containing nitrogen vacancy centers (NV), currently relying on pre‐screening diamond prior to fabrication of the AFM tips. Additionally, improvement of the signal counts in the in situ PL setup is feasible by cooling the sample, leading to an enhanced emission into the zero‐phonon line, in turn reducing the spectral integration window and therefore the background noise. To summarize, our technological approach of utilizing a FIB Poisson process of filling an array using in situ PL feedback potentially solves the most critical bottleneck for solid‐state quantum emitters, i.e., the creation process with simultaneous high‐yield and positional accuracy, to achieve proof‐of‐concept quantum photonic devices.^[^ ^16^, ^24^ ^]^

## 5 Experimental Section

### Sample Preparation

4H‐SiC was acquired from II‐VI Inc. with 10 µm epitaxial layer with 5 × 10^14^ cm^−3^ N impurity. The wafer was patterned with a 200 nm Al hard mask via e‐beam metal deposition. The wafer was patterned by standard photolithography. Then a BCl_3_/Cl_2_ dry etch in ICP was performed to remove Al followed by CF_4_/O_2_ chemistry to etch SiC to a depth of 680 nm. The patterned and etched wafer was then diced into pieces of size 5 × 5 mm^2^. The pieces were cleaned in Acetone/Isopropanol at 40 °C followed by Piranha (5:1 H_2_SO_4_:H_2_O_2_ at 100 °C), nitric acid (5:1:1 NH_4_OH:H_2_O_2_:H_2_O at 40 °C), and a 4 h soak in 1:1 49% HF:69% HNO_3_.^[^ ^13e^ ^]^ Since the cleaning procedure removed the Mo hard mask, the etch step was crucial for later identification of implant locations and FIB alignment.

### In Situ PL Setup

A 785 nm CW laser (Coherent OBIS LX) was used as an excitation source that passed through a dichroic beamsplitter and enters inside the vacuum chamber through an optical window. A high 0.9 NA objective microscope was used to excite and collect the PL from the sample. A widefield lens was used in the excitation path to increase the widefield spot size to about 10 µm. The collected PL passed through the same dichroic beamsplitter and a long pass filter of 830 nm. A tube lens focused the PL to the liquid nitrogen cooled CCD/spectrometer combination (Pylon 400BR/Acton SP2300i).

### Time‐Resolved Measurements

For time‐resolved photoluminescence lifetime and second‐order autocorrelation experiments, a picosecond pulsed 780 nm laser (Picoquant LDH 780‐B) with a repetition rate of 5 MHz was used for sample excitation and a Hanbury Brown‐Twiss setup consisting of a 50:50 beamsplitter and two single photon avalanche photodiodes (Excelitas SPCM‐AQRH‐14) with timing resolution of 300 ps were used. TRPL was analyzed using a TCSPC module (Picoquant Hydraharp 400).

## Conflict of Interest

The authors declare no conflict of interest.

## Supporting information

Supporting Information

## Data Availability

The data that support the findings of this study are available from the corresponding author upon reasonable request.
