# Supplementary material for: High‐Yield Deterministic Focused Ion Beam Implantation of Quantum Defects Enabled by In Situ Photoluminescence Feedback
Source: Adv Sci (Weinh). 2023 Apr 23;10(18):2300190. doi: 10.1002/advs.202300190 (PMC10288259; doi:10.1002/advs.202300190)
Supplement: Supplementary file 1 — Supporting Information [file ADVS-10-2300190-s001.pdf]

ADVANCED SCIENCE
Open Access

Supporting Information

for *Adv. Sci.*, DOI 10.1002/advs.202300190

High-Yield Deterministic Focused Ion Beam Implantation of Quantum Defects Enabled by In Situ Photoluminescence Feedback

Vigneshwaran Chandrasekaran, Michael Titze, Anthony R. Flores, Deanna Campbell, Jacob Henshaw, Andrew C. Jones, Edward S. Bielejec and Han Htoon**

Supporting Information for

High-Yield Deterministic Focused Ion Beam Implantation of Quantum Defects Enabled by In-Situ Photoluminescence Feedback

Vigneshwaran Chandrasekaran, [1]† *Michael Titze,*[2*]† *Anthony R. Flores,*[2] *Deanna Campbell,*[2] *Jacob Henshaw,*[3] *Andrew C. Jones,*[1] *Edward S. Bielejec,*[2] *Han Htoon*[1*]

[1] Center for Integrated Nanotechnologies, Materials Physics and Applications Division, Los Alamos National Laboratory, Los Alamos, NM 87545, USA

[2] Ion Beam Laboratory, Sandia National Laboratories, Albuquerque, NM 87123, USA

[3] Center for Integrated Nanotechnologies, Sandia National Laboratories, Albuquerque, NM 87123, USA

* E-mail: mictitz@sandia.gov & htoon@lanl.gov

† These authors contributed equally.

SI-A Sub-50 nm Targeting Resolution

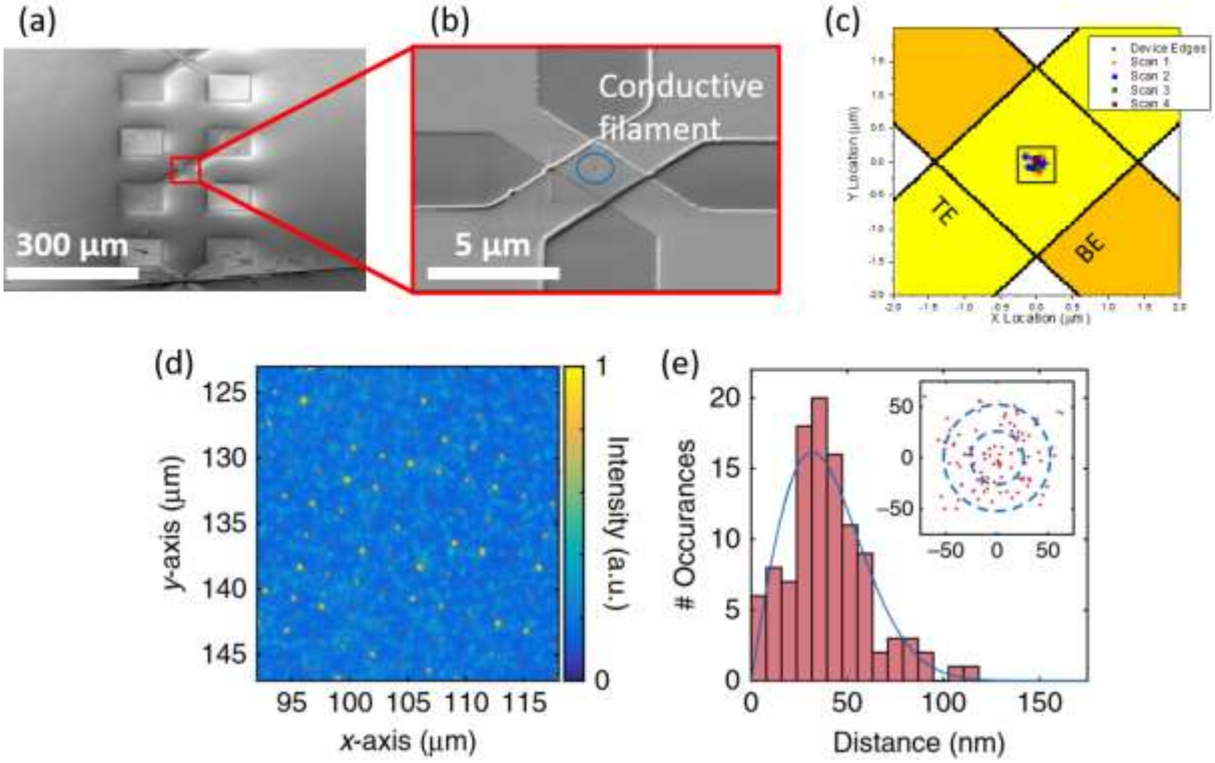

Figure SI-1. (a) HR-TEM image showing 200keV Si^{++} ion irradiation formed conductive filaments in memristor device. (b) Zoomed in view showing the conductive filament formed at the center of the device. (c) Ion beam induced charge collection map of implant locations in memristor device. The standard deviation of target positions is ±17 nm. (d) Photoluminescence map of 200 keV Si^{++} implantation formed silicon vacancy centers in diamond. (e) Histogram of color center location deviation from target position. The inset shows the spread of all implanted color centers with the one and two-standard deviation shown as the dashed blue lines.
Figure (c) adapated from [1], Figures (d) and (e) reproduced from [2]

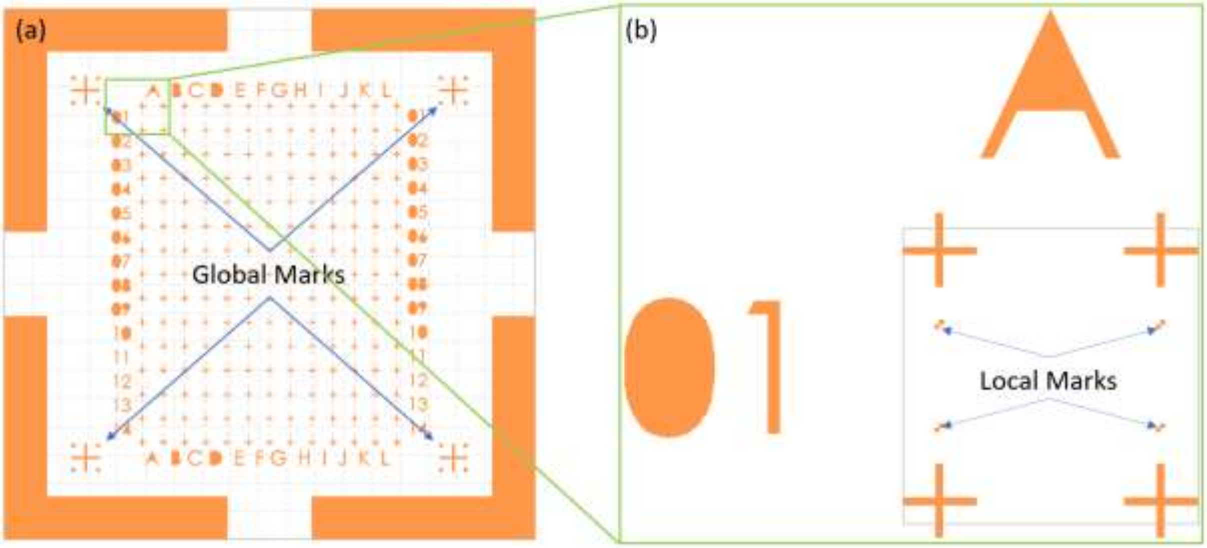

Figure SI-2. (a) Whole-chip overview of alignment marker pattern used for achieving sub-50 nm targeting resolution. Global marks are used to transform the stage coordinate system enabling sub-µm positioning accuracy and navigation based on GDS files. (b) Single working area view. A single 200 µm writefield is denoted by the gray box. The local marks are scanned with the ion beam used during experiments by secondary electron detection. Then the writefield is distorted, rotated and scaled accordingly by lithography software.

SI-B Second-order Autocorrelation

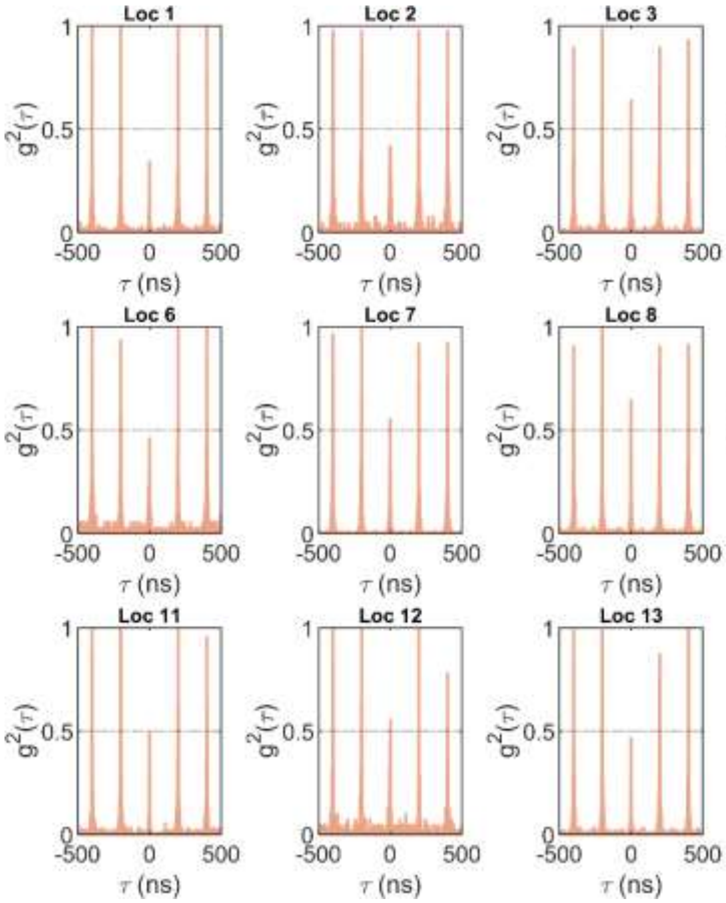

Figure SI-3. (a) Second-order autocorrelation $g^2(\tau)$ for all the 13 targets after background correction. 9 locations at 1, 2, 4, 5, 6, 9, 10, 11, and 13 show $g^2(0) < 0.5$. 4 locations at 3, 7, 8, and 12 show $g^2(0) > 0.5$ and < 0.7. The number of emitters created can be predicted by estimating $g^2(0) = 1 - 1/n$ for n number of emitters.[3] It is expected that $g^2(0) = 0$ for 1 emitter, $g^2(0) = 0.5$ for 2 emitters, $g^2(0) = 0.67$ for 3 emiters and so on. Therefore, we have 9 locations with single emitters and 4 locations with at most two emitters.

References:

[1] J. L. Pacheco, D. L. Perry, D. R. Hughart, M. Marinella, E. Bielejec, *Appl. Phys. A* **2018**, 124, 626.

[2] T. Schröder, M. E. Trusheim, M. Walsh, L. Li, J. Zheng, M. Schukraft, A. Sipahigil, R. E. Evans, D. D. Sukachev, C. T. Nguyen, J. L. Pacheco, R. M. Camacho, E. S. Bielejec, M. D. Lukin, D. Englund, *Nat. Commun.* **2017**, 8, 15376.

[3] a)R. Loudon, *The quantum theory of light*, Oxford, Clarendon Press, Oxford **1973**; b)A. Beveratos, S. Kühn, R. Brouri, T. Gacoin, J. P. Poizat, P. Grangier, *Eur. Phys. J. D* **2002**, 18, 191; c)S. Castelletto, F. A. Inam, S. I. Sato, A. Boretti, *Beilstein J. Nanotechnol.* **2020**, 11, 740.
